# Lipidomic and Instrumental Evaluation of a Melatonin-Based In & Out Strategy Versus Topical Treatment in Skin Aging: A Randomized Prospective Trial

**DOI:** 10.3390/metabo15010033

**Published:** 2025-01-09

**Authors:** Francesca Colombo, Stefano Alfano, Massimo Milani

**Affiliations:** Medical Department, Cantabria Labs Difa Cooper, 21042 Caronno Pertusella, Italy

**Keywords:** melatonin, “In & Out” strategy, anti-ageing, lipidomic analysis, cosmetic, skin, face, ceramides

## Abstract

This study aimed to evaluate the efficacy of a novel “In & Out” strategy, combining topical and oral melatonin supplementation, in managing skin aging compared to topical treatment alone. A randomized, prospective study was conducted on 39 healthy females aged 55–69 years. Participants were divided into two groups: one received both the topical formula and oral melatonin supplementation (Group A), while the other received a topical melatonin-based formula (Group B). Clinical evaluations included lipidomic analysis, skin moisturization, and wrinkle depth analysis at baseline and after 84 days. The addition of oral melatonin supplementation to the topical regimen led to improvements in the skin’s lipid profile and moisturization levels. These findings suggest that combining topical and oral melatonin may provide a more comprehensive approach to managing skin aging by addressing both local and systemic factors. **Background/Objectives**: With age, the endogenous antioxidant capacity of the skin decreases, including melatonin (Mel) synthesis. Skin aging is also associated with alterations in epidermal lipids, particularly a reduction in triglycerides and ceramides, which are essential for maintaining skin structure and hydration. The administration of exogenous melatonin could, therefore, be an effective anti-aging strategy. While some data suggest that melatonin may positively influence the lipid profile, specific data on its effects on skin aging are lacking. This study aimed to evaluate the anti-aging effects of an “In & Out” regimen consisting of a Mel-based cream and dietary supplement in comparison with topical treatment alone, focusing on clinical and lipidomic changes involved in skin homeostasis. **Results:** A statistically significant variation was observed in both groups compared to baseline (T0) in terms of moisturization (+23.6% in Group A, +18.3% in Group B) and wrinkle depth (−18.5% in Group A, −9.4% in Group B, *p* < 0.05). Both groups showed improvements in the lipid content of the skin, which typically decreases with age. The “In & Out” strategy resulted in a statistically significant increase in triacylglycerols and ceramides, key lipids that exhibit water-holding properties. **Conclusions**: The “In & Out” melatonin-based regimen demonstrated greater efficacy in clinical improvement and positive lipid profile modifications compared to topical treatment alone, highlighting its potential as a comprehensive anti-aging strategy.

## 1. Introduction

The aging of the skin is a natural and genetically determined process, influenced by both environmental (photoaging) and internal (Chrono aging) factors [1,2]. Skin aging determines progressive morphological and functional alterations like fine wrinkles, atrophy with reduced elasticity, and prominent dryness [1]. Chronological skin aging is caused mainly by an imbalanced endocrine circadian rhythmicity, with a hormonal decline and changes in gene expression [1,3]. The physiological aging at the skin level determines an increase in inflammation, mitochondrial dysfunction/ROS, and a decrease in antioxidant defenses inducing senescence of keratinocytes, fibroblasts, and melanocytes over time, contributing to decreased cutaneous regenerative potential [1]. Photoaging is determined by environmental stressors such as ultraviolet radiation (UV) and ambient pollutants, which stimulate reactive oxygen species (ROS) and reactive nitrogen species (RNS) production, and accumulation in the skin, generating oxidative stress. To counteract oxidative stress, the skin produces several protective molecules, including melatonin (Mel), vitamin D, and melanin [1,4,5,6]. Mel is an endogenous substance produced by the pineal gland but also by other tissues and organs such as the retina, gut, and skin [7]. Indeed, the skin is a complete and independent melatoninergic system and expresses Mel receptors MT1 (MTNRa) and MT2 (MTNRb) [8]. The functions of these receptors have been extensively studied over the years, confirming their roles in the cardiovascular, immune, and endocrine systems [9]. Additionally, they play a significant role in regulating skin pigmentation and aging, as well as in the production of vitamin D and protecting the skin against environmental damage [9,10]. Although Mel is best known for regulating circadian rhythmicity [11], it is involved in different physiological functions including skin functions, inflammatory response, and protection against oxidative stress [12,13]. Mel exerts protective action at the skin level against inflammation, oxidative stress, and mitochondrial damage [1]. The proprieties of Mel are in part related to its receptor-mediated action (cell growth regulation and skin tissue homeostasis) [14]. In addition, Mel exerts antioxidant proprieties directly, through its radical scavenging activity, and indirectly through its antioxidative enzyme-stimulating actions [12,15] and could also protect DNA from oxidative damage [14,16] and mitochondrial membrane potential [17,18]. Unfortunately, the endogenous antioxidant capacity of the skin is reduced with age, making the aged skin more vulnerable to environmental factors (e.g., UV radiation, air pollutants, and smoke). Therefore, exogenous melatonin administration could represent a good anti-aging strategy [1]. Thanks to its ability to easily penetrate the stratum corneum [19], the role of topical Mel as an anti-aging and photoprotective substance has been evaluated in different studies.

During aging, a change in the lipid profile of the skin occurs, including an alteration in the epidermal lipid component, with a reduction of lipids like triglycerides and ceramides, essential for maintaining skin structure and hydration. Some data suggest that Mel may have a positive effect on the lipid profile of the skin in terms of increasing ceramides and triglycerides. However, specific data on this effect on skin aging are limited. Du et al. demonstrated the efficacy of melatonin supplements in alleviating liver lipidome alteration by regulating endoplasmic reticulum stress in a mouse model [20].

The cream used in this study contains a specific technology (Melatonsphere^®^), where Mel is delivered into a mixture of two natural oils, Opuntia ficus indica and Persea gratissima seed oil, to improve the stability and skin penetration of Mel when used in topical formulations. The efficacy of this specific formulation was previously demonstrated by different clinical trials that observed the ability of the topical treatment to improve significantly skin tonicity and skin hydration with a significant reduction in skin roughness [8,21,22]. A recent study demonstrated that an “In & Out” regimen consisting of a Mel-based cream and a Mel-based (0.5 mg) dietary supplement, demonstrated a greater clinical improvement of skin aging signs in comparison with the topical treatment alone [23]. The oral hyaluronic acid could improve skin hydration, elasticity reducing transepidermal water loss, and facial wrinkles [24]. While apigenin, a natural flavone, is characterized by antioxidant and anti-inflammatory properties [25], and decreases the activity of MMP-1 [26]. Therefore, these activities could also contribute to the efficacy of the “In & Out” strategy.

Epidermal keratinocytes synthesize different lipids (e.g., cholesterol, free fatty acids, and ceramides) and are responsible for a correct epidermal permeability barrier. The aged skin displays a reduction in total lipid content compared to the young skin [27,28]. Among the lipids of the skin, triacylglycerols (TG), consisting of glycerol and three fatty acids (triesters), play an important role in skin homeostasis, contributing to the synthesis of ceramides (CER) important molecules for skin-barrier formation [29]. TG, with its increased chain length and unsaturation, seems to improve the CER content, fortifying the skin-barrier structure [29]. CERs are the most important lipids for skin-barrier homeostasis, and a decrease in the amount of CER is observed in aged skin, leading to a deficit in skin-barrier function and a consequent increase of transepidermal water loss (TEWL) [30]. CERs are characterized by a complex structure consisting of two major parts, a sphingoid base (SB) moiety and an amide-linked fatty acid (FA) moiety [29,31]. In the human stratum corneum, it is possible to find four main SB types, sphingosine [S], dihydrosphingosine [DS], phytosphingosine [P], and 6-hydroxy sphingosine [H] with three main FAs (non-hydroxy FA [N], a-hydroxy FA [A], and esterified ω-hydroxy FA [EO]) [29,32,33]. Skin lipids are “key components” involved in several functions and mechanisms in the skin, such as barrier function and microbiome composition. Therefore, it is important to evaluate and study their modifications.

Considering the literature, we wanted to evaluate the clinical efficacy of an “In & Out” regimen for skin-aging treatment, combining a Mel-based cream with a dietary supplement containing Mel (0.5 mg), hyaluronic acid (150 mg), and apigenin (0.9 mg), compared to topical treatment alone. Instrumental assessments of skin moisturization and wrinkle depth were used to confirm the superior effectiveness of the combined regimen. In addition, a lipidomic analysis was conducted to evaluate the role of these treatments in modulating the lipid content of the skin.

## 2. Materials and Methods

### 2.1. Population and Study Design

The study took place between February 2024 and June 2024. A total of 40 healthy female subjects, aged over 55 years (median age 59 years) with all skin types were enrolled and randomized in a 1:1 allocation ratio and instructed to apply the 0.1% Mel-based cream twice a day (Table 1).

They were also instructed to consume one tablet daily of a food supplement (Table 2) containing melatonin (0.5 mg/tablet), hyaluronic acid (150 mg/tablet), and apigenin (0.9 mg/tablet) (Group A, *n* = 20) or only to apply the cream twice a day (Group B, n = 20).

A dedicated computer program was used to generate the randomization list. Both products were commercially available (Cantabria Labs Difa Cooper, Caronno Pertusella, Italy). The study was conducted over 84 days, and participants were evaluated at baseline (T0) and after 84 days (T84).

### 2.2. Inclusion Criteria

The main inclusion criteria were healthy women aged >55 years, showing clinical signs of chrono-photoaging on the face as wrinkles and fine lines (type 3 on the Glogau Scale, assessed after clinical examination).

### 2.3. Exclusion Criteria

The main exclusion criteria were pregnancy or breastfeeding, allergy to components present in the products, and acute or chronic diseases able to interfere with the outcome of the study.

### 2.4. Instrumental Evaluation

The instrumental evaluations (skin moisturization and skin profilometry) were carried out in a temperature and humidity-controlled environment (respectively, T = 18–26 °C and RH = 50 ± 10%). The skin moisturization was evaluated using the Corneometer^®^ CM 825 (Courage + Khazaka, electronic GmbH, Cologne, Germany). The Corneometer^®^ measurement relies on assessing the capacitance of a dielectric medium, in this case, the stratum corneum—the outermost layer of the skin. As hydration increases, the dielectric properties of this layer change. This principle is based on the significantly higher dielectric constant of water (81) compared to most other substances, which generally have values below 7. The instrument generates an electric scatter field that penetrates the very first layers of the skin (10–20 μm). Changes in the dielectric constant resulted from variations in skin surface hydration, which affect the capacitance of a precision capacitor. This highly sensitive method can detect even minimal fluctuations in hydration levels. Unlike impedance-based techniques, it operates without a galvanic connection to the skin, avoiding any polarization effects. The PRIMOS optical system (GFMesstechnik GmbH, Berlin, Germany) was used to obtain three-dimensional (3D) measurements of the skin surface. This system operates by projecting a pattern of parallel stripes onto the skin’s surface using micro-mirrors integrated within a digital projector. A high-speed camera equipped with a sensitive optical sensor captures the deformed pattern. The recorded data are transmitted to a PC running the PRIMOS-CAD software v. 1.1 for analysis [34]. The system utilizes the principle of active image triangulation, which relies on the angle between the optical axes of the projector and camera lenses. Variations in the skin surface elevation cause distortions in the projected parallel stripes, enabling the generation of 3D topographical data. The software reconstructs a precise 3D image of the skin’s surface from the captured 2D images. This method allows for both qualitative and quantitative assessments of the skin profile, enabling rapid and accurate documentation of differences or changes in the surface structure [34]. Finally, for the lipidomic evaluation, skin-stripping samples were taken from the skin of the volunteers at baseline (T0) and after 84 days (T84) of treatment to verify how the treatments changed the composition and content of the skin surface lipids (SSLs) of the skin of the volunteers. The samples were stored at −80 °C until the day of analysis. Before the lipidomic analysis, each sample was extracted following a specific procedure [35].

### 2.5. Lipid Extraction

Samples were put into 2 mL polypropylene tubes. Internal standards were incorporated through 900 μL of methanol added into each tube. At 4 °C, the samples underwent agitation at a speed of 1400 rpm for an hour. The extracts obtained were then placed into an Amicon Multi-well Plate and dried in a SpeedVac concentrator. The dried residues were then washed by dissolving them in a solution containing 7.5 mM of ammonium acetate, chloroform, methanol, and propan-2-ol (1:2:4). To facilitate cholesterol analysis, an acetylation procedure followed by a drying process was carried out on the dried extract, after which the extract was dissolved in a similar mixture. To prevent droplet formation, a Hamilton Robotics STARlet was used to accomplish all the processes involved in applying liquids [35].

### 2.6. Liquid Chromatography

The extracted samples were analyzed using Agilent Technologies (Santa Clara, CA, USA) 1290 Infinity II liquid chromatograph UHPLC coupled to a 6560 Ion Mobility Q-TOF detector. For quality control, validation, and retention time, locking 69 deuterated standards from 14 lipids classes spiked into all samples are aligned, corrected, and used for signal intensity calibration and validation. Compound identification was achieved using four-dimensional data 4D-ID^®^ using a custom version of the lipidome atlas, MSDIAL 4.

### 2.7. Statistical Analysis

Statistical analyses were conducted using GraphPad Prism version 9.0 (GraphPad Software Inc., La Jolla, CA, USA). Instrumental data are submitted to 2-way ANOVA (intra-group analysis vs. T0 and inter-group statistical analysis Group A vs. Group B). Data are expressed as mean ± Standard Deviation (SD); variations are considered statistically significant when the *p*-value is <0.05. For lipidomic evaluation, the obtained results were subjected to statistical analysis using a Student *t*-test. Variations (vs. T0) are considered statistically significant with *p* < 0.05. Groups are compared to assess the significance and magnitude of differences in lipid expression by calculating *p*-values and fold changes. Fold changes (FC) quantify the relative change in expression levels between T0 and T84 and between groups, providing insight into the magnitude of biological differences. The FC between two groups is calculated as a simple ratio of the group means for a particular analyte.

## 3. Results

The clinical trial was carried out between February and June 2024, enrolling 40 healthy female participants with a mean age of 59 years (range: 55–69 years) (Table 3). All subjects, except one (2.5%), completed the treatment period.

**Figure 1:**
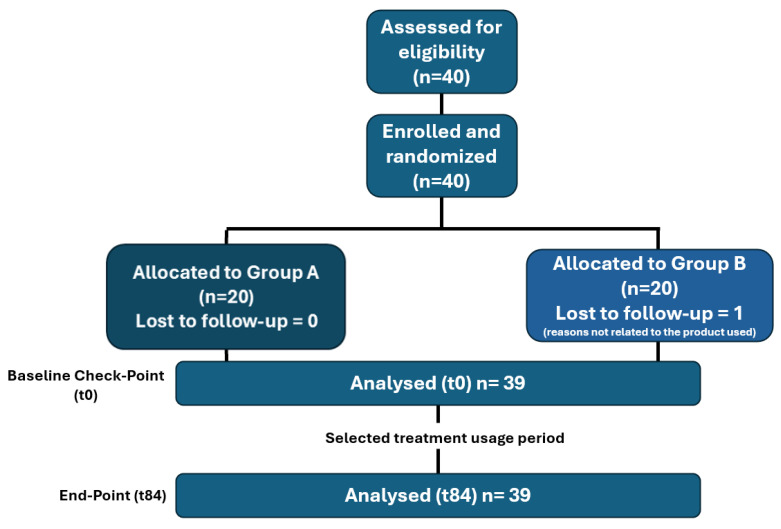
Flow diagram of the study.

Participants were randomized into two groups: Group A (n = 20, Mel-based cream + Mel-based oral supplement) and Group B (n = 19, Mel-based cream only). The flow diagram of the study is illustrated in Figure 1.

Skin moisturization at baseline (T0) was 43.3 ± 1.8 c.u. in Group A and 41.5 ± 1.7 c.u. in Group B. A statistically significant increase in skin moisturization at T84 compared to baseline was observed in both groups (+23.6% and +18.3% in Groups A and B, respectively).

**Figure 2:**
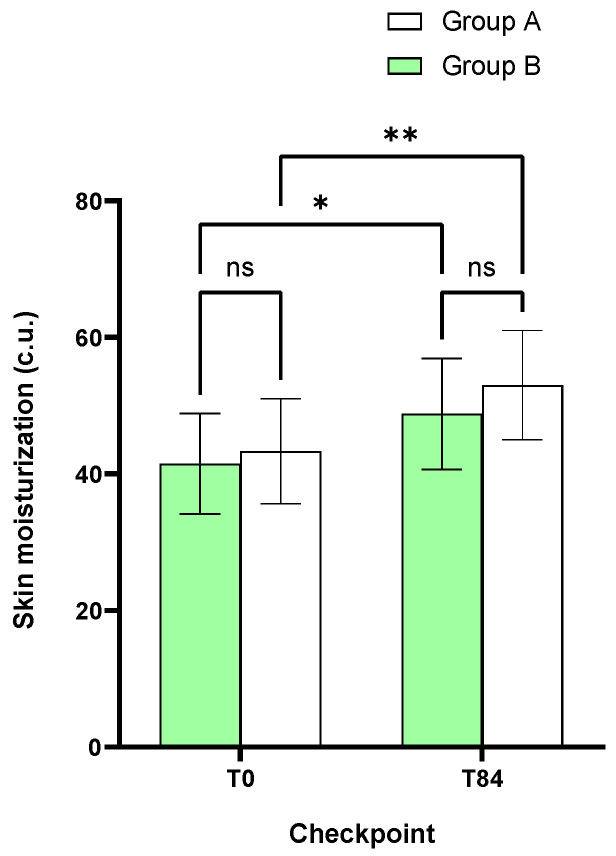
Mean data of the skin moisturization obtained at baseline (T0) and after 84 days (T84). Data are expressed as mean ± SD. Above the error bar the intra-group statistical analysis: * *p* < 0.05; ** *p* < 0.002.

No statistically significant variations between the two groups were highlighted for this parameter (Figure 2).

**Figure 3:**
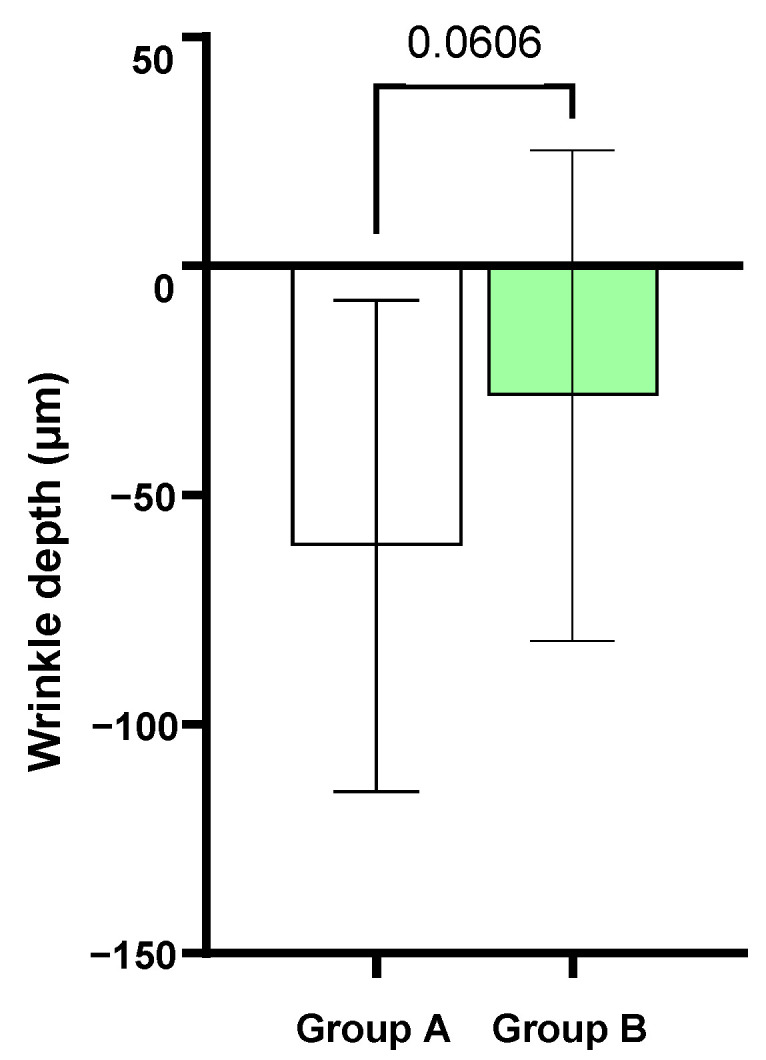
Absolute difference between Group A (Mel-based cream + Mel-based oral supplement) vs. Group B (Mel-based cream).

The skin profilometry was instrumentally evaluated using Primos 3D, which allowed us to evaluate the skin surface properties. In this trial, the wrinkle depth parameter was analyzed (Figure 3).

The wrinkle depth at baseline (T0) was 353.5 ± 30.0 μm in Group A and 339.0 ± 23.5 μm in Group B. The Mel-based cream combined with the Mel-based oral supplement resulted in an 18.5% reduction in wrinkle depth compared to T84. Similarly, the topical treatment alone (Group B) led to a 9.4% decrease in wrinkle depth compared to baseline at T84.

However, comparing the wrinkle depth of Group B vs. Group A, there was not a statistically significant difference between the two groups (*p* = 0.0606), but we could observe a tendency to decrease more in Group B compared to Group A.

At the end of the study (T84), volunteers were asked to express their opinions about tested products by answering a self-assessment questionnaire. Table 4 reports the items and the percentage of positive answers relative to the questionnaire.

The tested products were positively evaluated by most of the subjects enrolled for all the considered aspects. A higher percentage of positive responses (≥90%) was observed in Group A (Mel-based cream + Mel-based oral supplement). In both groups, 100% of the participants tolerated the tested products well.

**Figure 4:**
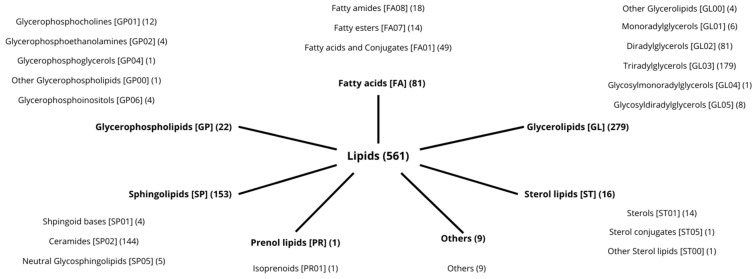
Graph of the lipid categories identified (numbers of molecules for each category).

The lipidomic analysis allowed us to identify and quantify the lipidic molecules that changed significantly in the two groups after 84 days of treatment. A total of 561 lipid molecules were considered. Molecules belonging to seven different lipid categories (fatty acyls, glycerolipids, sphingolipids, glycerophospholipids, sterol lipids, prenol lipids, and others) were divided into 22 Lipid Main Classes and 47 Lipid Subclasses (Figure 4).

Triacylglycerols (TG) play an important role in skin homeostasis. The Mel-based cream (Group B, Table 5) significantly increased the presence of triglycerides in the skin of volunteers.

In Group A (Mel-based cream + Mel-based oral supplement), the increase of TG was smaller compared to Group B (7 TG vs. 35 TG, Group A and B, respectively), but the amount of TG at T84 was still 21.54 times higher than at T0. Additionally, there was a statistically significant increase in the ceramide content in Group A compared to Group B, which could contribute to improved skin health by fortifying the skin-barrier structure [36].

Ceramides, members of the sphingolipid family, are not only the building blocks of epidermal barrier structure but also bioactive metabolites involved in epidermal self-renewal and immune regulation. In aged skin, studies have demonstrated that all major lipid species decreased, especially the ceramide content [36], indicating an alteration in the lipid barrier of the skin that leads to increased transepidermal water loss (TEWL) [37]. As reported in Table 5 and Table 6 of this study, the quantity of ceramides was particularly increased in Group A compared to the baseline.

In Group A, there was a statistically significant increase in Cer-EOS, the two primary subclasses of ceramides along with Cer-NS. These ceramides contribute to the formation of the corneocyte lipid envelope in the stratum corneum and play a critical role in establishing a functional epidermal permeability barrier [38].

The data in Table 5 indicates that in Group A, the EO-type ceramides, were the most abundant ceramide class that exhibited a significant increase compared to baseline (T0).

## 4. Discussion

Chronologically aged skin exhibits multiple functional changes in both the dermis and epidermis, including compromised permeability homeostasis, reduced stratum corneum hydration, and elevated skin-surface pH [27]. Additionally, external factors like UV radiation, smoke, and air pollutants promote mitochondrial dysfunction and oxidative damage due to excessive ROS generation, potentially leading to premature skin aging [1]. To counteract free radicals and oxidative stress, the skin produces protective molecules, including melatonin [1,6,39]. Melatonin (Mel), the main neuroendocrine secretory product of the pineal gland [11], is also produced by extra-pineal tissues like the skin. Human skin presents an efficient melatoninergic system and significant levels of melatonin [40,41].

While melatonin is known to regulate circadian rhythms, it also possesses potent antioxidants, DNA-protective, mitochondrial-protective, and anti-senescence activities. Unfortunately, endogenous melatonin synthesis decreases with aging, but exogenous melatonin can be used as an effective photoprotective [1,42,43] and promising anti-aging strategy [1,8]. The anti-aging efficacy of topical melatonin has been evaluated in clinical studies. Topical melatonin can penetrate the stratum corneum due to its low molecular weight and lipophilicity [44]. Additionally, the Mel used in the present study was incorporated in lipospheres (Melatonsphere^®^), further enhancing its skin penetration [8]. The clinical efficacy of this specific formulation has been previously demonstrated by various clinical trials, showing its ability to improve skin tonicity and hydration, and significantly reduce skin roughness [8,20,21]. Moreover, a recent clinical trial found that an “In & Out” regimen, consisting of a melatonin-based cream and a melatonin-based dietary supplement, containing hyaluronic acid and apigenin, resulted in greater clinical improvement of skin aging signs compared to topical treatment alone [23].

In this study, the clinical efficacy of the “In & Out” regimen, consisting of a Mel-based cream and a Mel-based dietary supplement containing hyaluronic acid and apigenin, was confirmed through instrumental evaluations of skin moisturization and wrinkle depth compared to the topical treatment alone. After 84 days, both groups showed a statistically significant increase in moisturization compared to baseline, although no statistically significant variations between groups were observed. Regarding skin profilometry, while neither group exhibited a statistically significant decrease in wrinkle depth compared to baseline at T84, a trend suggested more significant improvement in Group A patients compared to Group B. This data confirms the clinical efficacy of the Mel-based cream and the potential benefits of the “In & Out” strategy, which demonstrated greater clinical improvement of skin aging signs compared to the topical treatment alone.

Skin aging results in changes to baseline transepidermal water loss (TEWL). Various alterations can contribute to a compromised permeability barrier function. According to the “brick and mortar” model, the skin barrier is largely determined by the quality and quantity of differentiation-related proteins and extracellular lipids in the stratum corneum [27]. Epidermal keratinocytes synthesize cholesterol, free fatty acids, and ceramides are responsible for a proper epidermal permeability barrier. Studies have shown that the aged stratum corneum displays a >30% reduction in total lipid content compared to the young stratum corneum [27,28]. In this context, it is, therefore, important to evaluate the role of cosmetics or oral supplements in modulating the lipid content of the skin. Therefore, the secondary outcome of this study was to assess the skin lipidomic variation after 84 days of product use. Triacylglycerols are among the lipids that statistically increased in the treated groups, particularly in Group B compared to Group A. Although they are a minor component of the lipid matrix in the stratum corneum [45,46], they play a crucial role in skin homeostasis, as their metabolism is influential in epidermal differentiation and the skin-barrier function [45]. While the group using the Mel-based cream + Mel-based oral supplement showed a smaller increase in TG compared to the group using only the topical treatment, the fatty acid chains involved were longer. Longer chain length and increased unsaturation of TG appear to improve ceramide content, thereby fortifying the skin-barrier structure [29].

Ceramides are members of the sphingolipid family and constitute a critical hydrophilic extracellular lipid matrix that is indispensable for permeability barrier function. In addition, ceramides also act as active second messengers, regulating keratinocyte proliferation and differentiation, enhancing proinflammatory cytokine production, and modulating immune responses. Importantly, the ceramide content in the skin decreases with age. Both treatments used in this study significantly increased CER levels compared to baseline, particularly the “In & Out” strategy. Notably, only Group A showed a statistical increase in Cer-EOS and Cer-NP. CER-NP, with its phytosphingosine backbone, can form more hydrogen bonds with surrounding ceramides [47], which is crucial for modulating skin permeability barrier function [29]. CER-EO, containing an additional esterified fatty acid linked to an ultra-long ω-hydroxy fatty acid, plays a fundamental role in forming the corneocyte lipid envelope and maintaining skin-barrier structure and function [29].

## 5. Limitations

One notable limitation of our study is the absence of a placebo control group, which would have allowed for a more robust comparison of the treatment effect relative to a non-intervention group. Additionally, the relatively short duration of the experiment may have limited our ability to observe long-term outcomes or potential delayed effects of the intervention. Another limitation is the lack of diet control, as variations in participants’ dietary habits could influence skin health and potentially confound the results. However, despite these limitations, our study is assessor-blinded, minimizing the risk of bias in outcome evaluation. Furthermore, we employed objective instruments to assess patients’ skin, which enhances the reliability of the findings by reducing potential operator error or subjective bias. These strengths in our methodology provide confidence in the validity of our results, despite the constraints mentioned above. Anyway, future placebo-controlled studies could be useful for further confirmation of the results we have observed in this trial.

## 6. Conclusions

In agreement with previous clinical studies, the results of this study emphasize the significant benefits of combining topical Mel-based cream with an oral supplement for improving skin-barrier function and mitigating signs of skin aging. Both strategies led to a significant improvement in skin moisturization, and the “In & Out” strategy proved to be the most effective at increasing ceramide subclasses that are critical for skin-barrier function, such as Cer-EOS and Cer-NP. These findings indicate that the combination of topical and oral interventions not only enhances skin hydration and reduces wrinkle depth, but also promotes a more robust skin barrier by modulating the lipid content and fortifying ceramide profiles. This multifaceted approach, which addresses the problem of aging skin from both an external and internal perspective, has the potential to be an effective strategy for restoring permeability barrier function and revitalizing the skin.

## Figures and Tables

**Table 1:** List and amount of the ingredients of the melatonin-based cream.

Melatonin-Based Cream		
Ingredients	Quantity (%)	Function
Aqua	67.5	Solvent, base of the formulation
Butyrospermum Parkii Butter	5	Emollient, skin conditioning agent
Glycerin	5	Humectant, retains moisture
Cetearyl Alcohol	4	Emulsifier, thickener
Ethylhexyl Stearate	3.3	Emollient, improves skin texture
Caprylic/Capric Triglyceride	3	Emollient, skin conditioning
Glyceryl Stearate Citrate	3	Emulsifier, stabilizes the formulation
Isononyl Isononanoate	3	Emollient, skin conditioning
Rosa Moschata Seed Oil	1	Antioxidant, nourishing oil
Persea Gratissima Oil	0.9998	Moisturizer, skin conditioning agent
Phenethyl Alcohol	0.684	Preservative, antimicrobial agent
Tocopheryl Acetate	0.6	Antioxidant, protects skin from free radicals
Caprylyl Glycol	0.516	Preservative, antimicrobial agent
Opuntia Ficus Indica Seed Oil	0.5	Skin conditioning, antioxidant
Hydroxyethyl Acrylate/Sodium Acryloyldimethyl Taurate Copolymer	0.366	Thickener, stabilizer
Propanediol	0.36	Solvent, humectant
Ethylhexylglycerin	0.3	Preservative booster, humectant
Carbomer	0.18	Thickener, stabilizer
Polyisobutene	0.132	Film-forming agent, texture enhancer
Trisodium Ethylenediamine Disuccinate	0.111	Chelating agent, enhances preservative efficacy
Sodium DNA	0.1	Moisturizing, anti-aging agent
Pentaerythritol Tetra-di-t-butyl Hydroxyhydrocinnamate	0.1	Antioxidant, protects formulation stability
Melatonin	0.1	Active ingredient, antioxidant, skin repair
PEG 7 Trimethylolpropane Coconut Ether	0.042	Solubilizer, emulsifier
Lecithin	0.0408	Emulsifier, skin conditioning agent
Picea Abies Extract	0.04	Antioxidant, anti-inflammatory agent
Sodium Hydroxide	0.018	pH adjuster
Sorbitan Isostearate	0.018	Emulsifier, stabilizer
Tocopherol	0.0048	Antioxidant, protects skin and formulation
Ascorbyl Palmitate	0.004	Antioxidant, protects skin and formulation
Citric Acid	0.0005	pH adjuster, antioxidant

**Table 2:** List and quantity per tablet of the ingredients of the melatonin-based food supplement.

Melatonin-Based Cream		
Ingredients	Quantity (%)	Function
Aqua	67.5	Solvent, base of the formulation
Butyrospermum Parkii Butter	5	Emollient, skin conditioning agent
Glycerin	5	Humectant, retains moisture
Cetearyl Alcohol	4	Emulsifier, thickener
Ethylhexyl Stearate	3.3	Emollient, improves skin texture
Caprylic/Capric Triglyceride	3	Emollient, skin conditioning
Glyceryl Stearate Citrate	3	Emulsifier, stabilizes the formulation
Isononyl Isononanoate	3	Emollient, skin conditioning
Rosa Moschata Seed Oil	1	Antioxidant, nourishing oil
Persea Gratissima Oil	0.9998	Moisturizer, skin conditioning agent
Phenethyl Alcohol	0.684	Preservative, antimicrobial agent
Tocopheryl Acetate	0.6	Antioxidant, protects skin from free radicals

**Table 3:** List of the demographic details of the population (age, biological sex, and clinical signs on the face).

Demographic Details of the Population	
Age (mean, range)	59 years (range 55–69 years)
Biological Sex	Women
Clinical signs (on the face)	Clinical signs of chrono-photoaging on the face (wrinkles and fine lines)
Glogau Scale	Type 3

**Table 4:** Results of the self-assessment questionnaire expressed as a percentage of positive answers: % of subjects who gave positive judgment (completely agree or agree for items 01–08; very satisfied or satisfied for item 09; and Yes, for item 10).

Question Number	Survey Question	Positive Answers (%)	
		Group A	Group B
01	My skin is more hydrated	95	84.2
02	My skin is more nourished	95	84.2
03	My skin is softer	95	84.2
04	My skin appears more smoothed	90	84.2
05	My skin appears rejuvenated	90	78.9
06	The appearance of wrinkles and fine lines is attenuated	90	78.9
07	The skin is more radiant	95	78.9
08	The overall appearance of my skin is improved	95	84.2
Global appreciation			
09	Are you satisfied with the product/treatment?	95	89.5
10	Was the product/treatment well tolerated?	100	100

**Table 5:** List of molecules grouped for lipids classes that showed a statistically significant change between T0 and T84 in Group B (Mel-based cream), fc = fold change.

Mel-Based Cream (Group B)			
Lipid Class	Numbers of Lipids per Class	Fc	*p*-Value
TG	35	147.5	0.021
DG	16	37.91	0.016
MG	2	8.61	0.019
NAE	2	4.04	0.013
PI	2	5.02	0.039
Cer_HS	1	5.02	0.027
DGCC	1	1.11	0.032
DGTS	1	2.45	0.007
EtherTG	1	2.43	0.004
FAHFA	1	0.33	0.049
NABAGA	1	1.73	0.023
Others	1	0.28	0.011
OxTG	1	4.29	0.01

**Table 6:** List of molecules grouped for lipids classes that showed a statistically significant change between T0 and T84 in Group A (Mel-based cream + Mel-based oral supplement), fc = fold change.

Mel-Based Cream + Mel Based Oral Supplement (Group A)			
Lipid Class	Numbers of Lipids per Class	Fc	*p*-Value
Cer_EOS	9	13.77	0.018
DG	8	21.73	0.029
TG	7	21.54	0.03
OxTG	5	22.38	0.028
NAE	4	5.56	0.014
DGCC	2	1.67	0.026
Cer_HS	2	2.61	0.02
Cer_EODS	1	1.48	0.004
Cer_HDS	1	1.28	0.032
Cer_NP	1	1.42	0.003
Cer_NS	1	1.32	0.039
EtherMGDG	1	4.5	0.017
EtherTG	1	2.87	0.045
MG	1	4.14	0.002
VAE	1	0.81	0.013
WE	1	1.48	0.015

## Data Availability

The data that support the findings of this study are available on request from the corresponding author. The data is not publicly available due to privacy or ethical restrictions.

## Abbreviation List

Abbreviation	Definition
Cer_EOS	Ceramide Esterified Omega-hydroxyacyl Sphingosine
DG	Diacylglycerol
TG	Tri-acylglycerol
OxTG	Oxidized Triacylglycerol
NAE	N-acylethanolamine
DGCC	Diacylglycerylcar-boxyalkylcholine
Cer_HS	Ceramide containing Hydroxyl Sphingosine
Cer_EODS	Ceramide Esterified Omega-hydroxyacyl Dihydroxy Sphingosine
Cer_HDS	Ceramide containing Hydroxy Dihydroxy Sphingosine
Cer_NP	Ceramide containing Non-Hydroxy Phytosphingosine
Cer_NS	Ceramide containing Non-Hydroxy Sphingosine
EtherMGDG	Ether-linked Monoacylglyceryl-Dihexosylglycerol
EtherTG	Ether-linked Triacylglycerol
MG	Monoacylglycerol
VAE	Vinyl Ether Acylglycerol
WE	Wax Esters
PI	Phosphatidylinositol
DGTS	Diacylglyceryltrimethylhomoserine
NABAGA	N-acetyl biotinyl-aminopropylglycine ascorbate
